# Increased sympathetic tone contributes to cardiovascular dysfunction in sepsis

**DOI:** 10.1186/cc12980

**Published:** 2013-11-05

**Authors:** Ana Maria Favero, Regina Sordi, Geisson Nardi, Jamil Assreuy

**Affiliations:** 1 Department of Pharmacology, Universidade Federal de Santa Catarina, Florianópolis, Brazil; 2 Department of Biological Sciences and Health, Universidade do Oeste de Santa Catarina, Joaçaba, Brazil

## Background

The cardiovascular dysfunction of sepsis/septic shock is characterized by hypotension, tachycardia/bradycardia, endothelial dysfunction and hyporesponsiveness to vasoconstrictors. Hypotension and low tissue perfusion trigger an increase in sympathetic tone probably as an attempt to restore blood pressure to normal levels. The persistently higher sympathetic stimulation may lead to the exhaustion of the capacity of vascular response and thus create a vicious circle contributing to vascular hyporesponsiveness and higher adrenergic stimulation. In addition, in septic shock patients, increased arterial levels of norepinephrine (NE) were significantly associated with mortality. The aim of this work was to evaluate the vascular response to an adrenergic agonist during severe sepsis and the effects of the early inhibition of sympathetic tone in sepsis-induced cardiovascular dysfunction.

## Materials and methods

Sepsis was induced by cecal ligation and puncture (CLP) surgery in female Wistar rats. Septic animals and controls (CT) were treated with the ganglionic blockers pentolinium (PENT; 5 mg/kg, s.c.) or hexamethonium (HEX, 15 mg/kg, s.c.) or vehicle (saline) 1 hour after surgery. The vascular response to the administration of NE was assessed 6 hours or 24 hours after CLP surgery. The survival rate was also evaluated. All procedures were approved by our Institutional Ethics Committee (PP00631/CEUA-UFSC) and are in accordance with NIH Animal Care Guidelines.

## Results

Six hours after CLP surgery, septic animals were hypotensive. Treatment with hexamethonium or pentolinium prevented the development of hypotension (control 84.8 ± 2.6; CLP 60.7 ± 4.5*; CLP + HEX 72.7 ± 3.1; CLP + PENT 78.1 ± 2.7 mmHg; *P < 0.05 compared with control group). However, 24 hours after surgery, the ganglionic blockers failed to prevent hypotension (control 88.5 ± 1.7; CLP 62.7 ± 1.7*; CLP + HEX 68.8 ± 2.7*; CLP + PENT 70.9 ± 3.4* mmHg; *P < 0.05 compared with control group). The vascular hyporesponsiveness to NE observed both 6 hours and 24 hours after CLP was completely blocked by the early treatment with both ganglionic blockers (NE 10 nmol/kg, expressed as increase in blood pressure compared with baseline: control 54.2 ± 4.5; CLP 6 hours 21.9 ± 3.1*; CLP 6 hours + HEX 52.6 ± 7.0; CLP 6 hours + PENT 54.1 ± 4.9; CLP 24 hours 31.1 ± 5.6*; CLP 24 hours + HEX 74.6 ± 3.0; CLP 24 hours + PENT 64.4 ± 7.8 mmHg; *P < 0.05 compared with control group). The early ganglionic blockade with PENT decreased the mortality observed after 96 hours.

## Conclusions

Our data indicate that increased sympathetic tone in sepsis contributes, at least in part, to the development of hypotension, hyporesponsiveness to vasoactive agents and mortality. Blockade of increased sympathetic tone thus may be considered as an adjuvant therapy for the treatment of septic cardiovascular dysfunction.

